# 

**Published:** 1896-01-11

**Authors:** 


### CONTENTS.

| | |
|---|---|
| **Latency of Infection** | 243 |
| **The Place of Research in Education** | 244 |
| **On Modern Progress in Ophthalmic Medicine and Surgery.** By ROBERT BRUDENELL CARTER, F.R.C.S.—Squint | 245 |
| **Modern Sociology.**—Self-help and Charitable Aid | 246 |
| **Annotations.**—The British Medical Association and Quackery—Sanitation means Clean Living—Nerves and Noises | 247 |
| **Notes and News** | 248 |
| **Scraps and Gleanings** | 257 |
| **The Student of Medicine** | 257 |
| **Medical Progress and Hospital Clinics—** | |
| The Schott Treatment for Heart Disease | 249 |
| PROGRESS IN MEDICINE.—Infective Diseases | 250 |
| PROGRESS IN SURGERY.—The Surgery of the Chest | 251 |
| Diseases of the Upper Air Passages | 253 |
| NEW APPLIANCES AND THINGS MEDICAL | 254 |
| **The Institutional Workshop—** | |
| HOSPITAL CONSTRUCTION.—The Floating Hospital on the River Tees | 255 |
| HOSPITAL SECRETARIES AND THEIR SALARIES | 256 |

### EXTRA SUPPLEMENT.—THE NURSING MIRROR

| | |
|---|---|
| **News from the Nursing World.**—A Christmas Gift—Nurses Controlled by the Laity—What was the Aggravation?—"Suggestion and Sympathy"—The Society of Trained Masseuses—Bolingbroke House Hospital—A Second Nurse Needed—"Only One or Two"—Glasgow Royal Infirmary—Accidentally Poisoned—A Nurse's Recognition in France—Dr. Wynter Blyth at Toynbee Hall—English Nurses for Johannesburg—Nurses in Miniature—Short Items | cxvii |
| **Lectures on Nursing.** IV.—Care of Bedding, Beds, Bed-pans, and Lavatories | cxix |
| **Nurses and their Competitors** | cxx |
| **Dress and Uniforms** | cxxi |
| **Medico-Psychological Association** | cxxii |
| **Christmas Entertainments** | cxxiii |
| **The Winter Exhibitions** | cxxiii |
| **Appointments** | cxxiv |
| **Presentations** | cxxiv |
| **Wants and Workers** | cxxiv |
| **Sleep and Sleeplessness** | cxxiv |
| **Royal British Nurses' Association** | cxxiv |
| **Everybody's Opinion** | cxxv |
| **New Registration of Nurses** | cxxv |
| **Notes and Queries** | cxxv |
| **For Reading to the Sick** | cxxvi |
| **Where to Go** | cxxv |
| **The Book World for Women and Nurses** | cxxvi |

